# A prospective, multi‐center, randomized comparison of iron isomaltoside 1000 versus iron sucrose in patients with iron deficiency anemia; the FERWON‐IDA trial

**DOI:** 10.1002/ajh.25564

**Published:** 2019-07-13

**Authors:** Michael Auerbach, David Henry, Richard J. Derman, Maureen M. Achebe, Lars L. Thomsen, John Glaspy

**Affiliations:** ^1^ Department of Medicine Georgetown University School of Medicine Washington DC; ^2^ Department of Medicine, Division of Hematology and Oncology Pennsylvania Hospital Philadelphia Pennsylvania; ^3^ Thomas Jefferson University Philadelphia Pennsylvania; ^4^ Brigham and Women's Hospital Dana Farber Cancer Institute, Harvard Medical School Boston Massachusetts; ^5^ Department of Clinical and Non‐clinical Research Pharmacosmos A/S Holbaek Denmark; ^6^ Department of Medicine, Division of Hematology Oncology UCLA School of Medicine Los Angeles California

## Abstract

Iron deficiency anemia (IDA) is prevalent, and intravenous iron, especially if given in a single dose, may result in better adherence compared with oral iron. The present trial (FERWON‐IDA) is part of the FERWON program with iron isomaltoside 1000/ferric derisomaltose (IIM), evaluating safety and efficacy of high dose IIM in IDA patients of mixed etiologies. This was a randomized, open‐label, comparative, multi‐center trial conducted in the USA. The IDA patients were randomized 2:1 to a single dose of 1000 mg IIM, or iron sucrose (IS) administered as 200 mg intravenous injections, up to five times. The co‐primary endpoints were adjudicated serious or severe hypersensitivity reactions, and change in hemoglobin from baseline to week eight. A total of 1512 patients were enrolled. The frequency of patients with serious or severe hypersensitivity reactions was 0.3% (95% confidence interval: 0.06;0.88) vs 0.4% (0.05;1.45) in the IIM and IS group, respectively. The co‐primary safety objective was met, and no risk difference was observed between groups. The co‐primary efficacy endpoint of non‐inferiority in hemoglobin change was met, and IIM led to a significantly more rapid hematological response in the first two weeks. The frequency of cardiovascular events was 0.8% and 1.2% in the IIM and IS group, respectively (*P* = .570). The frequency of hypophosphatemia was low in both groups. Iron isomaltoside administered as 1000 mg resulted in a more rapid and more pronounced hematological response, compared with IS, which required multiple visits. The safety profile was similar with a low frequency of hypersensitivity reactions and cardiovascular events.

## 1 INTRODUCTION

Intravenous (IV) iron is commonly used for the treatment of iron deficiency anemia (IDA) as an alternative to oral iron.^1^, ^2^, ^3^ Historically, IV iron has been associated with a number of safety concerns, including the potential for inducing oxidative stress, cardiovascular toxicity, and hypersensitivity reactions.^4^ The European Medicines Agency has officially raised concerns about rare hypersensitivity reactions following IV iron administration. Published evidence suggests that the overwhelming majority of acute infusion reactions are mild and are not due to hypersensitivity but rather complement activation.^5^, ^6^ These minor reactions usually consisting of pressure in chest or back or flushing are self‐limited and do not require intervention.^7^, ^8^ Unfortunately due to fears of anaphylaxis, inappropriate intervention with vasopressors and antihistamines convert minor reactions to serious adverse events, ostensibly attributed to the IV iron. Clinical trials evaluating iron safety are often limited by small numbers not statistically powered to detect small differences in rare hypersensitivity reactions.^9^ The FIRM trial included 1997 IDA patients and provided a direct comparative assessment of incidences of hypersensitivity reactions and hypotension associated with ferumoxytol and ferric carboxymaltose (FCM), reporting incidences of moderate‐to‐severe hypersensitivity reactions, including anaphylaxis, or moderate‐to‐severe hypotension of 0.6% and 0.7% in the ferumoxytol and FCM groups, respectively.^10^

IV iron has been shown to be beneficial in heart failure with guidelines now recommending IV iron to this population.^11^ However, some IV iron formulations bind the elemental iron less tightly with subsequent higher amounts of labile free iron,^12^ which is toxic and causes oxidative stress and cell damage,^13^ playing a vital role in the mechanisms behind cardiovascular diseases.^14^ Thus, IV iron formulations with a carbohydrate binding the iron more tightly resulting in slower release, allow much higher dosing in a significantly shorter period of time mitigating the preponderance of side effects due to free/labile iron.

The FERWON program with iron isomaltoside 1000/ferric derisomaltose (IIM) was initiated in order to evaluate the safety and efficacy of IIM and iron sucrose (IS) with a focus on serious or severe hypersensitivity reactions. Other IV iron products available in the US and EU are characterized by a structure with an iron core surrounded by a carbohydrate shell. By contrast, IIM has a unique defined matrix structure with alternating iron molecules and linear isomaltoside 1000 oligomers. The resulting matrix contains about 10 iron molecules per isomaltoside oligosaccharide in a strong and stably bound structure that enables a controlled and slow release of bioavailable iron to iron binding proteins with little risk of free iron toxicity.^12^ As a result, it can be administered in high doses and previous published clinical trials demonstrate good safety and efficacy of IIM in various populations with different comparators.^15^, ^16^, ^17^, ^18^, ^19^, ^20^, ^21^, ^22^, ^23^, ^24^, ^25^, ^26^, ^27^ The FERWON program consists of two trials including 3050 IDA patients with either different clinical diagnoses (FERWON‐IDA trial) or chronic kidney disease (FERWON‐NEPHRO trial). Herein we present the results of the FERWON‐IDA trial. The aim was to evaluate the safety and efficacy of IIM and iron sucrose (IS) in a broad population with IDA.

## 2 METHODS

### 2.1 Trial design

This was a prospective, comparative, open‐label, randomized, multi‐center trial. For each individual, duration of the trial was approximately 10 to 15 weeks. All attended at least six visits (a screening visit, a baseline visit including investigational product administration, three assessment visits at week one, two, and four, and a final visit at week eight). If no documented intolerance of oral iron was present for at least one month, within the last nine months prior to enrolment, a run‐in period with oral iron for up to one month to document intolerance or lack of response to oral iron was required. One to four additional visits during the run‐in period (one telephone visit to initiate the run‐in period, two telephone visits to assess compliance and tolerance, and one visit to assess compliance, tolerance, and response) were scheduled. Those randomized to IS attended two additional visits, if deemed necessary to achieve the cumulative dose of IS required.

The protocol and amendments were approved by the relevant Institutional Review Boards and conducted in accordance with good clinical practice and the Declaration of Helsinki of 1975, as revised in 2008. The trial was registered with ClinicalTrials.gov (NCT02940886). Written informed consent was obtained from all participants.

### 2.2 Participants

The trial was conducted at 114 sites in the USA. Patients were ≥18 years with IDA of different etiologies, and had intolerance or lack of response to oral iron or screening hemoglobin (Hb) measurement sufficiently low to require rapid repletion of iron stores. Patients with Hb ≤11 g/dL, transferrin saturation (TSAT) <20%, and *s*‐ferritin <100 ng/mL, were allowed to participate in the trial, after having signed the informed consent form. Inclusion and exclusion criteria are shown in Table S1.

### 2.3 Interventions

Randomization was 2:1 to two groups. Onewas IIM (Monofer/Monoferric, Pharmacosmos A/S, Holbaek, Denmark) administered at baseline, as a single dose of 1000 mg infused over 20 minutes. The other was iron sucrose (Venofer, American Regent, Shirley, New York, USA) administered as 200 mg IV injections according to label and repeated up to five times (a cumulative dose of 1000 mg was recommended). During the trial, other iron supplementation than the investigational drug, blood transfusion, and erythropoiesis stimulating agents were prohibited.

### 2.4 Objectives and endpoints

The trial was designed with the objectives to evaluate and compare safety and efficacy of IIM to IS in patients with IDA, when oral iron formulations were ineffective or could not be used, or where there was a clinical need to deliver iron rapidly.

The co‐primary endpoints were number with adjudicated serious or severe hypersensitivity reactions, starting on or after the first dose of treatment, and change in Hb from baseline to week eight. The secondary safety endpoints included adjudicated composite cardiovascular adverse events (AEs), starting on or after the first dose of randomized treatment, and the incidence of hypophosphatemia (*s*‐phosphate <2.0 mg/dL), which was measured at all site visits. The secondary efficacy endpoints included Hb increase of ≥2 g/dL and *s*‐ferritin level of ≥100 ng/mL and TSAT of 20% to 50%. Also included were changes in Hb, *s*‐ferritin, TSAT, and fatigue symptoms measured by the Functional Assessment of Chronic Illness Therapy (FACIT) Fatigue Scale.

Adjudication of serious or severe hypersensitivity reaction and composite cardiovascular AEs was performed in a blinded fashion by an independent Clinical Endpoint Adjudication Committee. The hypersensitivity terms were defined by a standardized set of Medical Dictionary for Regulatory Activities (MedDRA) terms, which are listed in Table S2. The terms were based on discussions with FDA.

### 2.5 Sample size

The significance level was set to 5%. With N = 1000 in the IIM treatment group, the power was 88% for demonstrating that the upper boundary of the 95% confidence interval (CI) of the incidence of treatment emergent for serious or severe hypersensitivity reactions, was less than 3%. The incidence rate of 3% was based on the incidences reported for the same endpoint on FCM and IS^28^ The upper boundary of the 95% CI for the incidence of serious or severe hypersensitivity reaction, should be less than 3% (2 × 1.5%) in the IIM treatment group, in order to meet the co‐primary safety objective. With N = 500 in the IS group, assuming no difference between the treatment groups, and assuming a common SD (SD) of 1.5 g/dL, the power was 100% for demonstrating non‐inferiority of the change in Hb from baseline to week eight, using a non‐inferiority margin of −0.5 g/dL. This yielded a total power of 88% for demonstrating both co‐primary endpoints.

### 2.6 Randomization

A stratified block randomization methodology was used in the trial, and randomization was a 2:1 ratio to receive IIM or IS, respectively. Randomization was stratified according to the type of underlying disease (gastroenterology, gynecology, oncology, and “other”), and baseline cardiovascular risk (history of myocardial infarction, stroke, or congestive heart failure). The block size was six.

### 2.7 Data analysis sets

**Figure 1:**
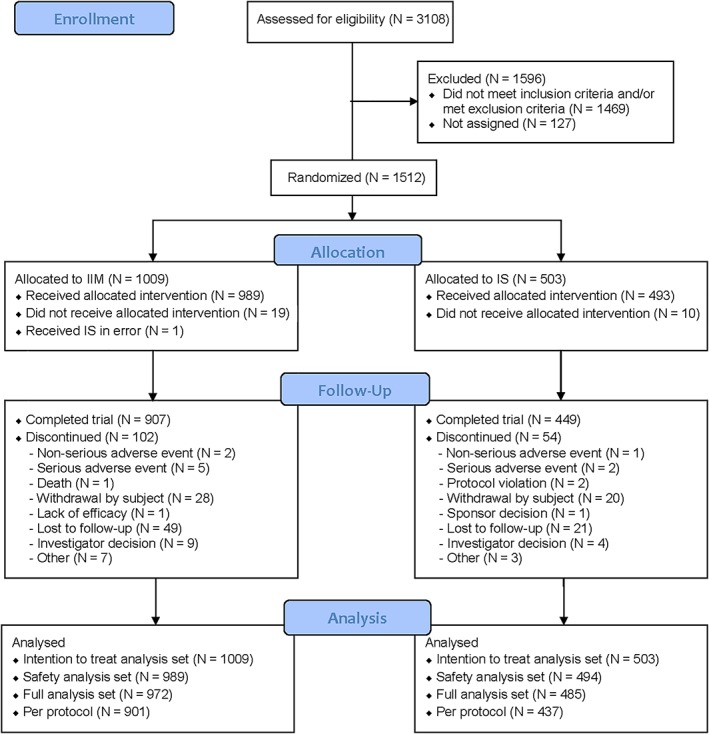
Patient disposition. IIM, iron isomaltoside 1000/ferric derisomaltose; IS, iron sucrose

The following data sets were used in the analyses (Figure 1).

The intention to treat (ITT) analysis set (N = 1512) included all randomized. This was used for evaluating efficacy.

The safety analysis set (N = 1483) included all randomized who received at least one dose of the trial drug. This was used for evaluating safety.

The full analysis set (FAS; N = 1457) included all randomized patients who received at least one dose of the trial drug, and had at least one post‐baseline Hb assessment. This was used for a sensitivity analysis of the co‐primary efficacy endpoint.

The per protocol (PP) analysis set (N = 1338) included all in the FAS who did not have any major protocol deviations of clinical or statistical significance. This was used for a sensitivity analysis of the co‐primary efficacy endpoint.

### 2.8 Statistical analyses

The co‐primary safety endpoint, serious or severe hypersensitivity reaction, was summarized, and if the upper boundary of the 95% CI was <3%, the safety objective was met. In addition, as supportive information, the risk difference between IIM and IS was assessed by constructing a 95% CI of the risk difference. The overall incidence of composite cardiovascular AEs, and frequency of patients with adverse drug reactions (ADRs), were compared between the treatment groups by a Fisher's exact test.

The co‐primary efficacy endpoint, change in Hb from baseline to week eight, was tested for non‐inferiority by using a mixed model for repeated measurements (MMRM) with a restricted maximum likelihood (REML)‐based approach. The model included the fixed, categorical effects of treatment, week, treatment‐by‐week interaction, strata, and the continuous covariates of baseline Hb and baseline Hb‐by‐week interaction. Non‐inferiority of IIM against IS could be claimed if the lower boundary of the 95% CI was above −0.5 g/dL.

The secondary efficacy endpoints were tested for superiority. Incidence of Hb responders to each week (defined as an increase in Hb of at least 2 g/dL from baseline to the week in question) was analyzed using a repeated measures logistic regression model with treatment, visit, strata, and treatment by visit interaction as fixed effects and baseline value as covariate.

Time to Hb response was estimated using the Kaplan‐Meier method. The hypothesis of no treatment difference was assessed by a two‐sided log‐rank test.

The incidence of patients who achieved a *s*‐ferritin level of ≥100 ng/mL and a TSAT of 20‐50% at any time were analyzed using a logistic regression model with treatment and strata as fixed effects.The MMRM, with treatment, week, treatment‐by‐week interaction, and strata as factors and baseline value and baseline value‐by‐week interaction as covariates, was used to compare the average change in Hb, *s‐*ferritin, TSAT, and fatigue symptoms.

All tests were two‐tailed and the significance level was 0.05. The baseline characteristics and other safety data (including incidence of hypophosphatemia) were displayed descriptively.

Patients and investigators were not blinded to trial medications during the trial. However, the hypersensitivity reactions and cardiovascular AEs were evaluated centrally by a blinded independent adjudication committee. Also, laboratory parameters were evaluated at a central laboratory, so it was not deemed necessary to have a blinded trial design.

## 3 RESULTS

### 3.1 Patients

A group of 3108 patients were screened of whom 1512 were randomized 2:1 to the IIM group (1009) or IS group (503). Of the 1512 enrolled, 1356 (90%) completed the trial. The details of patient disposition are summarized in Figure 1.

Baseline demographics and laboratory parameters are summarized in Table 1. Overall baseline characteristics were comparable between the treatment groups. Approximately 50% were gynecology patients and 26% were gastroenterology patients.

**Table 1:** Summary of baseline demographics, hemoglobin, *s*‐ferritin, transferrin saturation, and *s*‐iron (intention to treat analysis set)

	IIM	IS
Age (years)		
N	1009	503
Mean (SD)	44.1 (14.8)	43.8 (14.4)
Min;max	18;91	18;91
Gender (N [%])		
Women	892 (88.4)	456 (90.7)
Men	117 (11.6)	47 (9.3)
Race (N [%])		
White	504 (50.0)	264 (52.5)
Asian	8 (0.8)	4 (0.8)
Black or African American	484 (48.0)	223 (44.3)
American Indian or Alaska Native	4 (0.4)	1 (0.2)
Native Hawaiian or other Pacific Islander	1 (0.1)	2 (0.4)
Other	8 (0.8)	9 (1.8)
Ethnicity (N [%])		
Hispanic or Latino	387 (38.4)	205 (40.8)
Not Hispanic or Latino	622 (61.6)	298 (59.2)
Hemoglobin (g/dL)		
N	1009	503
Mean (SD)	9.25 (1.28)	9.17 (1.27)
Min;Max	4.0;13.5	5.2;13.8
*S*‐ferritin (ng/mL)		
N	1009	503
Mean (SD)	14.4 (42.6)	11.9 (37.6)
(Min;max)	1;729	1;715
Transferrin saturation (%)		
N	1009	503
Mean (SD)	7.43 (10.93)	6.69 (7.44)
(Min;max)	1;176	1;84

*Note*: Some patients had baseline hemoglobin, transferrin saturation, and s‐ferritin values that were increased above the inclusion screening values (hemoglobin ≤11 g/dL, transferrin saturation < 20%, and s‐ferritin <100 ng/mL).

Abbreviations: %, percentage of patients; IIM, iron isomaltoside 1000/ferric derisomaltose; IS, iron sucrose; Max, maximum; Min, minimum; N, number of patients; SD, standard deviation.

### 3.2 Exposure to iron

A total of 989 patients received IIM, and 494 patients received IS. One infusion in the IIM group, and one to five infusions in the IS group were administered, with the majority receiving five (80%; mean: 4.5 administrations, median: 5 administrations). The mean dose for IIM and IS was 975 mg (SD: 145), and 905 mg (SD: 217), respectively.

### 3.3 Serious or severe hypersensitivity reactions ‐ co‐primary safety endpoint

The safety analyses were conducted on the safety analysis set (N = 1483).

A total of three serious or severe hypersensitivity reactions were reported in 3/989 (0.3%; 95% CI: 0.06;0.88) patients in the IIM group, and two serious or severe hypersensitivity reactions were reported in 2/494 (0.4%; 95% CI: 0.05;1.45) in the IS group. As the upper boundary of the 95% CI was <3%, the co‐primary safety endpoint was met. The risk difference between IIM and IS, with respect to adjudicated and confirmed treatment emergent serious or severe hypersensitivity reactions was estimated to −0.10% (95% CI: −0.91;.71). The difference between the two treatment groups was not statistically significant as the confidence interval included zero. Narratives of the hypersensitivity reactions are provided in Table S3.

### 3.4 Composite cardiovascular adverse events

Eight composite cardiovascular AEs were reported in eight (0.8%) patients in the IIM group, and seven were reported in six (1.2%) in the IS group. The incidence of composite cardiovascular AEs was, however, not statistically significantly different between the two treatment groups (*P >* .05).

### 3.5 Hypophosphatemia

The incidence of hypophosphatemia (*s*‐phosphate <2.0 mg/dL) was low and similar in the two groups (3.9% in the IIM and 2.3% in the IS group).

The hypophosphatemia events were transient, and in most cases normalized at the end of the trial. For the majority, the lowest *s‐*phosphate values were reached at week one or two.

The incidence of severe hypophosphatemia (*s*‐phosphate <1.0 mg/dL) was 0.0% in both groups, as no patient had a *s*‐phosphate level < 1.0 mg/dL during the trial.

### 3.6 Adverse drug reactions and other safety data

A total of 230 ADRs in 124 patients (12.5%) were reported in the IIM group, and 138 ADRs in 63 patients (12.8%) were reported in the IS group (*P >* .05). The most common ADR was nausea (20 events in 20 patients [2.0%] in the IIM group and 10 events in 8 patients [1.6%] in the IS group). Rash (17 ADRs in 15 patients [1.5%]) and chest discomfort (11 ADRs in 11 patients [1.1%]) were reported more frequently in the IIM group than in the IS group (0 ADR and 0 ADR, respectively). Dysgeusia (20 ADRs in 9 patients [1.8%]) and overdose (10 ADRs in 8 patients [1.6%]), were more common in the IS group than in the IIM group (1 and 0 ADRs, respectively).

One in the IIM group died three months after treatment with IIM in a hospice. The event was reported as worsening of pre‐existing cancer, in a patient with underlying bile duct cancer, and was not related to the trial drug.

### 3.7 Change in hemoglobin

The co‐primary efficacy analysis (change in Hb concentration from baseline to week eight) was conducted on the ITT (N = 1512), FAS (N = 1457), and PP analysis set (N = 1338), and the secondary efficacy analyses were conducted on ITT.

**Figure 2:**
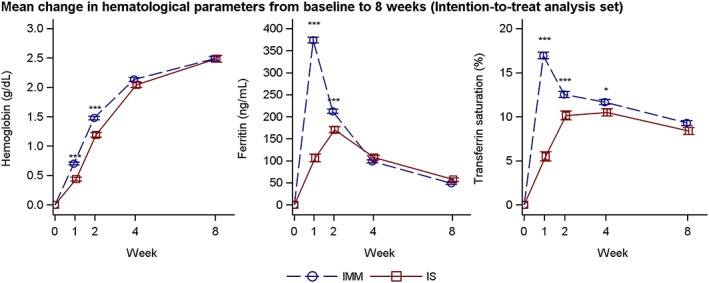
Hemoglobin, *s*‐ferritin, and transferrin saturation over time by treatment group (intention to treat analysis set). Estimates (mean and SE) from a mixed model with repeated measures with strata, treatment and time as factors, treatment*time and baseline value*time interactions and baseline value as covariate. IMM, iron isomaltoside 1000/ferric derisomaltose; IS, iron sucrose. *P < .05, **P < .001, ***P < .001

The increase in Hb concentration from baseline to weeks one and two was statistically significantly greater for IIM compared with IS (*P* < .0001). The increase from baseline to week four did not differ statistically significantly between treatment groups (*P* = .109) (Figure 2).

A summary of the co‐primary efficacy analysis is provided in Table 2. The change in Hb concentration from baseline to week eight was non‐inferior for IIM compared to IS since the lower boundary of the 95% CI for the treatment difference (IIM‐IS) was above −0.5 g/dL. Superiority of IIM vs IS could not be claimed since the 95% CI included zero. This was confirmed in all three analysis sets (Table 2).

**Table 2:** Analysis of change in hemoglobin (g/dL) from baseline to week eight

Treatment	N	LS Mean [95% CI]	Difference IIM—IS Estimate [95% CI]	Non‐Inferior^a^	Superiority test *P*‐value
Intention to treat analysis set					
IIM	1009	2.49 [2.41;2.56]	0.00 [−0.13;0.13]	Yes	0.977
IS	503	2.49 [2.38;2.59]			
Full analysis set					
IIM	972	2.51 [2.43;2.58]	0.01 [−0.12;0.14]	Yes	0.834
IS	485	2.49 [2.39;2.60]			
Per protocol analysis set					
IIM	901	2.58 [2.50;2.65]	0.01 [−0.12;0.14]	Yes	0.871
IS	437	2.57 [2.46;2.68]			

Abbreviations: CI, confidence interval; IIM, iron isomaltoside 1000/ferric dersisomaltose; IS, iron sucrose; LS Mean, Least square mean.

a Non‐inferiority could be claimed if the lower boundary of the 95% CI is above −0.5 g/dL.

The proportion of responders (Hb increase of ≥2 g/dL from baseline) was statistically significantly higher in the IIM group, compared to the IS group at weeks one and two, but not at weeks four or eight (Table S4). The median time to Hb increase of ≥2 g/dL was 28 days in both the IIM and the IS groups (*P* = .088).

### 3.8 Change in s‐ferritin and transferrin saturation

The proportion with *s‐*ferritin ≥100 ng/mL and TSAT of 20% to 50% at any time from weeks one to eight was statistically significantly higher in the IIM group compared with the IS group (70% vs 34%, *P* < .0001).

The increase in *s*‐ferritin from baseline to weeks one and two was statistically significantly greater for IIM compared with IS (*s*‐ferritin: *P <* .0001 at both weeks), while the increase from baseline to weeks four and eight did not differ statistically significantly between treatment groups (Figure 2).

The increase in TSAT from baseline to weeks one, two, and four was statistically significantly greater for IIM compared with IS (*P* < .0001, *P* = .0001, and *P* = .016), while the increase from baseline to week eight did not differ statistically significantly between treatment groups (Figure 2).

### 3.9 Change in fatigue symptoms

At baseline, more than half had severe fatigue (a FACIT fatigue score <30 indicates severe fatigue) as indicated by a median FACIT fatigue score of 24.0 in both treatment groups. The mean FACIT fatigue score increased from baseline to week eight in both treatment groups (IIM group: from 25.72 to 39.98; IS group: from 24.63 to 39.93). The increase in FACIT fatigue score from baseline to week one was significantly greater for IIM compared with IS (*P* = .042). The increase from baseline to weeks two and eight did not differ statistically significantly between treatment groups (Figure S1).

## 4 DISCUSSION

The objectives of this trial were to evaluate the safety with special focus on the risk of hypersensitivity reactions, and efficacy of IV IIM, in comparison to the widely used IS in a broad population with different IDA etiologies. Iron sucrose was chosen as comparator in the trial, as iron sucrose has consistently shown a low risk of hypersensitivity in clinical trials.^29^ The IDA etiology was in approximately 50% of cases a gynecological disorder, and in 26% a gastroenterological disorder. Here, IDA was confirmed in all based on low values of Hb, TSAT, and *s*‐ferritin at screening (Hb ≤11 g/dL, TSAT <20%, and *s*‐ferritin <100 ng/mL). The mean cumulative dose was 975 mg (SD: 145) and 905 mg (SD: 217) for IIM and IS, respectively. The cumulative doses reflect the difference in dosing opportunities between the two IV iron products, where IIM allows administration of the full iron dose in one visit, whereas IS requires multiple visits to reach the same dose level. All except one received the full IIM dose, whereas only 80% received the recommended IS dose.

Three serious or severe hypersensitivity reactions in three (0.3%) patients were positively adjudicated in the IIM group, and two reactions in two (0.4%) patients in the IS group. The 95% CI for the percentage reporting serious or severe hypersensitivity reactions in the IIM treatment group was 0.06;0.88. As the upper boundary was <3%, the co‐primary safety objective was met, and no risk difference to IS was observed. The frequencies of serious or severe hypersensitivity reactions are lower than those published in a previous review of IV irons.^30^ One explanation for this difference could be that the hypersensitivity reactions reported in the review were not adjudicated and confirmed by an independent Clinical Endpoint Adjudication Committee, as in the present trial. In the FIRM trial, where moderate‐to severe hypersensitivity reactions, including anaphylaxis and hypotension, were assessed for ferumoxytol and FCM, the frequencies were 0.6% and 0.7%, respectively.^10^ These reactions were also adjudicated by a blinded and independent Clinical Event Committee, however, the terms used for defining a hypersensitivity event were different compared to our trial.

When comparing IIM with IS, there was no statistically significant difference in the number with composite cardiovascular AEs (0.8% vs 1.2%) in this relative low risk population. A pre‐specified pooled analysis with the data from the FERWON‐NEPHRO trial will determine whether IIM has an advantage over IS in this regard.

The number with ADRs (12.5% vs 12.8%) was similar, where rash and chest discomfort were only reported in the iron isomaltoside group, whereas dysgeusia was reported more frequently in the iron sucrose group. These side effects are already known side effects consistent with the well‐established safety information approved by regulatory authorities for both compounds.

The frequency of hypophosphatemia (*s‐*phosphate <2.0 mg/dL) (3.9% vs 2.3%) in both groups was low, and none had a *s‐*phosphate <1.0 mg/dL. Thus, hypophosphatemia was not a safety issue in this trial, which is supported by previous trials with IIM showing a low frequency of hypophosphatemia in different populations.^31^

Non‐inferiority was claimed for change in Hb from baseline to week eight for IIM compared with IS, and treatment with IIM led to a higher increase in Hb at weeks one and two. This was supported by the proportion of responders (Hb increase of ≥2 g/dL from baseline), which was statistically significantly higher in the IIM group compared to the IS group at weeks one and two. The faster response was also shown for *s*‐ferritin, and TSAT, and it is likely due to the higher doses of IIM given within a shorter time period. The faster efficacy response observed with IIM is consistent with a previously reported trial performed with IIM and IS in an IDA population.^27^

Both IIM and IS led to an increase in FACIT fatigue score with a significant difference at week one in favor of iron isomaltoside. Thus, treatment of IDA with IIM may not only lead to a correction of the hematology parameters but may also increase QoL by decreasing symptoms such as fatigue faster since IIM has a shorter treatment period to reach the clinically required iron dose. In a sub‐analysis of women suffering from severe fatigue after postpartum hemorrhage, a single dose of IIM was associated with a statistically significant and clinically relevant reduction in aggregated physical fatigue within 12 weeks after delivery, when compared to current treatment practice with oral iron.^32^

In conclusion, IV IIM administration was well tolerated in a broad population with IDA being intolerant or unresponsive to oral iron therapy or being in need of iron rapidly. The number with serious or severe hypersensitivity reactions was low for IIM with no risk difference to IS and hypophosphatemia was not a safety issue for IIM or IS. Administration of a single dose of IV IIM provided a faster and transiently greater Hb response within the first 2 weeks and was as effective in ensuring an improvement in Hb concentration at 8 weeks as IV IS given up to five doses over 2 weeks. This trial contributes to the body of evidence demonstrating that IIM is safe and efficacious in treatment of IDA in a single visit.

## CONFLICT OF INTERESTS

Michael Auerbach receives research funding for data management from AMAG Pharmaceuticals.

David Henry has no conflict of interest.

Richard Derman has been a research and science consultant with Pharmacosmos and AMAG.

Lars L. Thomsen is employed by Pharmacosmos A/S.

Maureen M. Achebe has been on scientific advisory boards for Pharmacosmos and AMAG pharmaceuticals, and a consultant for Luitpold.

John Glaspy has been an advisor to AMAG Pharmaceuticals.

This work was funded by Pharmacosmos A/S and the investigators/institutions received a fee per patient.

## Supporting information


**Table S1** Inclusion and exclusion criteria
**Table S2** Hypersensitivity terms defined by a standardized set of Medical Dictionary for Regulatory Activities (MedDRA) terms
**Table S3** Narrative of serous or severe hypersensitivity reactions
**Table S4** Analysis of proportion of patients with hemoglobin increase of ≥2 g/dL from baseline to weeks one, two, four, or eight (intention to treat analysis set)
**Figure S1** Change in fatigue symptoms (FACIT fatigue symptoms) from baseline to weeks one, two, and eight (intention to treat analysis set)
