# Supplementary material for: A prospective, multi‐center, randomized comparison of iron isomaltoside 1000 versus iron sucrose in patients with iron deficiency anemia; the FERWON‐IDA trial
Source: Am J Hematol. 2019 Jul 13;94(9):1007–14. doi: 10.1002/ajh.25564 (PMC6772897; doi:10.1002/ajh.25564)
Supplement: Supplementary file 1 — Table S1 Inclusion and exclusion criteria Table S2 Hypersensitivity terms defined by a standardized set of Medical Dictionary for Regulatory Activities (MedDRA) terms Table S3 Narrative of serous or severe hypersensitivity reactions Table S4 Analysis of proportion of patients with hemoglobin increase of ≥2 g/dL from baseline to weeks one, two, four, or eight (intention to treat analysis set) Figure S1 Change in fatigue symptoms (FACIT fatigue symptoms) from baseline to weeks one, two, and eight (intention to treat analysis set) [file AJH-94-1007-s001.pdf]

Supplementary tables

Table S1 Inclusion and exclusion criteria

Inclusion criteria:

1. Men or women ≥18 years
2. Subjects having iron deficiency anemia (IDA) caused by different aetiologies* such as abnormal uterine bleeding, gastrointestinal diseases, cancer, bariatric procedures (gastric bypass operations), and other conditions leading to significant blood loss
3. Subjects with:
   a. Up to 1 month run-in phase indicating intolerance or lack of response to oral iron or
   b. A documented history of intolerance to oral iron therapy to at least one month of ordinated oral iron therapy within 9 months** prior to trial enrolment or
   c. Screening hemoglobin (Hb) measurement in Investigators' opinion was sufficiently low as to require rapid repletion of iron stores to minimise the risk of eventual blood transfusion***
4. Hb ≤11 g/dL
5. Transferrin saturation <20%
6. S-ferritin <100 ng/mL
7. Willingness to participate and signing the informed concent form

*The aetiology for IDA was to be documented in the medical history and verified in the source document.

**The intolerance to oral iron treatment within the last 9 months was to be documented in the medical history and verified in the source document with signs and symptoms that led to discontinuation of the oral iron therapy and precluded further use of oral iron therapy.

*** The Hb measurement that in Investigators' opinion was sufficiently low as to require rapid repletion of iron stores to minimise the risk of receiving a blood transfusion was to be documented in the source document.

After the run-in period, subjects who fulfilled the following additional eligibility criteria were included:

1. Hb level ≤11 g/dL
2. S-ferritin ≤800 ng/mL
3. Lack of efficacy: Hb increase <1 g/dL at visit R4 (i.e. inadequate response to oral iron) and compliance to oral iron (pill counts), which needed to be ≥67%
   Or
4. Hb level ≤11 g/dL
5. S-ferritin ≤800 ng/mL
6. Inability to tolerate oral iron, as per discretion of the Investigator

Exclusion criteria:

1. Anaemia predominantly caused by factors other than IDA according to Investigator's judgment
2. Haemochromatosis or other iron storage disorders
3. Known hypersensitivity reaction to any component of iron isomaltoside 1000/ferric derisomaltose (IIM) or iron sucrose (IS)
4. Previous serious hypersensitivity reactions to any intravenous iron compounds

5. Previously randomised in a clinical trial with IIM
6. Received an investigational drug within 30 days of screening
7. During 10-day period prior to screening; has been treated with intravenous iron
8. Erythropoiesis stimulating agent treatment within 30 days before the screening visit
9. During 30-day period prior to screening or during the trial period; has or will be treated with a red blood cell transfusion, radiotherapy, and/or chemotherapy
10. During 30-day period prior to screening or during the trial period; has or will require a surgical procedure that necessitated general anaesthesia
11. Alanine aminotransferase and/or aspartate aminotransferase >3 times upper limit of normal (e.g. decompensated liver cirrhosis or active hepatitis)
12. Any non-viral infection
13. Required dialysis for treatment of chronic kidney disease
14. Alcohol or drug abuse within the past 6 months
15. Estimated life expectancy of <6 months or, for cancer patients, an Eastern Cooperative Oncology Group performance status >1
16. Any other laboratory abnormality, medical condition, or psychiatric disorders which, in the opinion of the Investigator, will put the subject’s disease management at risk or may result in the subject being unable to comply with the trial requirements
17. Pregnant or nursing women. In order to avoid pregnancy, women of childbearing potential have to use adequate contraception (e.g. intrauterine devices, hormonal contraceptives, or double barrier method) during the whole trial period and 7 days after the last dosing

Table S2 Hypersensitivity terms defined by a standardized set of Medical Dictionary for Regulatory Activities (MedDRA) terms

**Group A**	**Group B**	**Group C**
Anaphylactic reaction	Acute respiratory failure	Allergic oedema
Anaphylactic shock	Asthma	Angioedema
Anaphylactic transfusion reaction	Bronchial oedema	Erythema
Anaphylactoid reaction	Bronchospasm	Eye oedema
Anaphylactoid shock	Cardio-respiratory distress	Eye pruritus
Circulatory collapse	Chest discomfort	Eye swelling
First use syndrome	Choking	Eyelid oedema
Kounis syndrome	Choking sensation	Face oedema
Shock	Circumoral oedema	Flushing
Type I hypersensitivity	Cough	Generalised erythema
	Cyanosis	Injection site urticaria
	Dyspnoea	Lip oedema
	Hyperventilation	Lip swelling
**Group D**	Laryngeal dyspnoea	Ocular hyperaemia
Blood pressure decreased	Laryngeal oedema	Oedema
Blood pressure diastolic decreased	Laryngospasm	Periorbital oedema
Blood pressure systolic decreased	Laryngotracheal oedema	Pruritus
Cardiac arrest	Mouth swelling	Pruritus allergic
Cardio-respiratory arrest	Nasal obstruction	Pruritus generalised
Cardiovascular insufficiency	Oedema mouth	Rash
Diastolic hypotension	Oropharyngeal spasm	Rash erythematous
Hypotension	Oropharyngeal swelling	Rash generalised
	Respiratory arrest	Rash pruritic
	Respiratory distress	Skin swelling
	Respiratory failure	Swelling
**Group E**	Reversible airways obstruction	Swelling face
Loss of consciousness	Sensation of foreign body	Urticaria
Seizure	Sneezing	Urticaria papular
Syncope	Stridor	
Unresponsiveness	Swollen tongue	
	Tachypnoea	
	Throat tightness	
	Tongue oedema	
	Tracheal obstruction	
	Tracheal oedema	
	Upper airway obstruction	
	Wheezing	

Table S3 Narrative of serous or severe hypersensitivity reactions

**Patient A: Hypersensitivity reaction (iron isomaltoside)**<br>This case concerned a patient in the 30's, who experienced hypersensitivity. The patient was randomised to the iron isomaltoside group and received investigational product (1000 mg) at the baseline visit. At the time of entry into the trial, the patient had a medical history of penicillin allergy. The patient did not take any concomitant medication. The patient came for the baseline visit and iron isomaltoside infusion was started at 10:45. At approximately 10:50, the patient complained of shortness of breath and tightness in the chest. Infusion of iron isomaltoside was terminated, and the patient was placed on normal saline. At 10:51, vital signs showed blood pressure of 143/92 mmHg, heart rate of 103 bpm, and oxygen saturation of 94 %. The patient was administered 50 mg hydrocortisone sodium succinate and placed on 2 litres oxygen via nasal cannula. At 11:04, the patient reported relief of symptoms. Vital signs showed blood pressure of 128/88 mmHg, heart rate of 73 bpm, and oxygen saturation of 99 %. The patient recovered on the day of the baseline visit and went home. The Investigator assessed the event of hypersensitivity to be serious, severe, and related to iron isomaltoside. The event was adjudicated as a moderate hypersensitivity event.
**Patient B: Hypersensitivity reaction (iron isomaltoside)**<br>This case concerned a patient in the 50's, who experienced hypersensitivity. The patient was randomised to the iron isomaltoside group and received investigational product (90 mg) at the baseline visit. At the time of entry into the trial, the patient had a medical history of hypercholesterolaemia, arthritis, cerebrovascular accident, and bronchial asthma. No relevant concomitant medication was reported. At the baseline visit, 1 minute after initiation of iron isomaltoside infusion, the patient complained of nausea, vomiting, and abdominal cramping. Vital signs were stable, oxygen saturation was 99-100 %, and ECG was normal. Infusion of iron isomaltoside was immediately terminated. The symptoms persisted and the patient was taken to the hospital for evaluation and management. Upon further questioning, the patient stated that the patient had received IV iron supplementation from another site approximately one month ago. This was not revealed to the site during the screening process. The patient recovered 2 days after the baseline visit and was discharged. The Investigator assessed the event of hypersensitivity to be serious, moderate, and possibly related to iron isomaltoside. The event was adjudicated as a severe hypersensitivity event.<br>Nausea and abdominal pain were symptoms of hypersensitivity for patient B. Both events were reported as AEs and adjudicated and confirmed as hypersensitivity events. As the events were symptoms of hypersensitivity, they have not been considered separate serious or severe hypersensitivity reactions.
**Patient C: Asthma exacerbation (iron isomaltoside)**<br>This case concerned a patient in the 50's, who experienced acute asthma exacerbation. The patient was randomised to the iron isomaltoside group and received investigational product (1000 mg) at the baseline visit. At the time of entry into the trial, the patient had a medical history of asthma, IDA, and depression. Concomitant medication included folic acid, ferrous sulfate and vitamin B complex. 8 days after the baseline visit, the patient was admitted to the hospital for acute asthma exacerbation. The patient presented with fatigue, shortness of breath, productive cough, and wheezing. The patient was treated with nebuliser, methylprednisolone sodium succinate, and levofloxacin. The patient recovered 9 days after the baseline visit and

was subsequently discharged on steroids, nebulisers and antibiotics on the same day. 22 days after the baseline visit, the patient was re-admitted for asthma exacerbation (recorded as a second SAE). The Investigator assessed the event of acute asthma exacerbation to be serious, moderate, and not related to investigational product. The event was adjudicated as a severe hypersensitivity event.
**Patient D: Anaphylactic reaction (iron sucrose)**<br><br>This case concerned a patient in the 20's, who experienced an anaphylactic reaction. The patient was randomised to the iron sucrose group and received investigational product at the baseline visit (200 mg) and 2 (200 mg) and 7 (200 mg) days later. At the time of entry into the trial, the patient had a medical history of heavy menstrual bleeding, IDA, and oral iron intolerance. The patient did not take any concomitant medication. At the last treatment visit, the patient received the third dose of 200 mg IV iron sucrose. Approximately 15 minutes later, the patient reported discomfort, muscle cramps, and showed a generalised skin rash. 125 mg methylprednisolone sodium succinate was administered intramuscularly, and approximately 10 minutes later the rash disappeared and the patient reported feeling better. While the patient was in observation, the patient started to sweat profusely, and reported skin cold and generalised tingling sensation. The patient's blood pressure fell from 115/71 mmHg to 90/50 mmHg. The rescue was called and they arrived shortly, where most of the symptoms had disappeared and the patient reported feeling better. However, during the recognition the paramedics noticed the patient showed signs of orthostatic hypotension and they decided to transfer the patient to the emergency room. Therapy with iron sucrose was withdrawn and the patient was discontinued from the trial. The patient recovered on the day of the last treatment. The Investigator assessed the event of anaphylactic reaction to be serious, moderate, and related to iron sucrose. The event was adjudicated as a severe hypersensitivity event.
**Patient E: Anaphylactic reaction (iron sucrose)**<br><br>This case concerned a patient in the 50's, who experienced an anaphylactic reaction following accidental overdose of iron sucrose. The patient was randomised to the iron sucrose group and received the first dose of investigational product (1000 mg) at the baseline visit. At the time of entry into the trial, the patient had a medical history of high blood pressure, thyroid disorder, and depression. The patient had no history of asthma, allergy, or eczema. Concomitant medication included aripiprazole, enalapril, escitalopram, levothyroxine, lovastatin, insulin aspart, and vitamin D. At the baseline visit, the patient received an IV bolus of iron sucrose at 12:18. Mistakenly, 1000 mg iron sucrose was administered instead of 200 mg. At 12:37, the patient stated that the patient felt nauseous. Normal saline was put to gravity and the doctor paged to infusion. At 12:38, vital signs showed blood pressure of 157/89 mmHg, heart rate of 93 bpm, and oxygen saturation of 96 %. At 12:39 the patient complained of tightness in the chest and 100 mg hydrocortisone sodium succinate was ordered. At 12:40, vital signs showed blood pressure of 122/78 mmHg, heart rate of 90 bpm, and oxygen saturation of 95 %. Oxygen was ordered at 2 litres via nasal cannula. At 12:42, the patient stated no relief of symptoms. At 12:44, blood pressure was unable to be assessed and heart rate not palpable. The patient was alert but lethargic. The doctor ordered 0.3 mg epinephrine via EpiPen. At 12:50, an ambulance was called to the office and at 13:00 the patient was taken to the emergency room but not admitted. In the emergency room, the patient was treated with Benadryl and was discharged after monitoring on the same day. The outcome of the event was recovered on the day of the baseline visit. The Investigator assessed the events of anaphylactic reaction and accidental overdose to be serious, severe, and related to iron sucrose. The event of anaphylactic reaction

was adjudicated as a severe hypersensitivity event. The event of accidental overdose was not referred for potential adjudication.

Table S4 Analysis of proportion of patients with hemoglobin increase of ≥2 g/dL from baseline to weeks one, two, four, or eight (intention to treat analysis set)

	**IIM**<br>**E/N (%)**	**IS**<br>**E/N (%)**
**Week 1**		
Responders	51/960 (5.3)	12/477 (2.5)
Odds ratio[a] [95% CI]	2.44 [1.27;4.72]	
P-value	0.008	
**Week 2**		
Responders	297/912 (32.6)	94/452 (20.8)
Odds ratio[a] [95% CI]	2.42 [1.80;3.26]	
P-value	<0.0001	
**Week 4**		
Responders	514/903 (56.9)	250/450 (55.6)
Odds ratio[a] [95% CI]	1.23 [0.96;1.58]	
P-value	0.105	
**Week 8**		
Responders	606/903 (67.1)	309/454 (68.1)
Odds ratio[a] [95% CI]	1.05 [0.80;1.38]	
P-value	0.703	

[a]IIM/IS

CI, confidence interval; E, number of responders; IIM, iron isomaltoside 1000/ferric derisomaltose; IS, iron sucrose; N, number of patients

Figure S1 Change in fatigue symptoms (FACIT fatigue symptoms) from baseline to weeks one, two, and eight (intention to treat analysis set)

Mean change in FACIT Fatigue Scale from baseline to 8 weeks (Intention-to-treat analysis set)

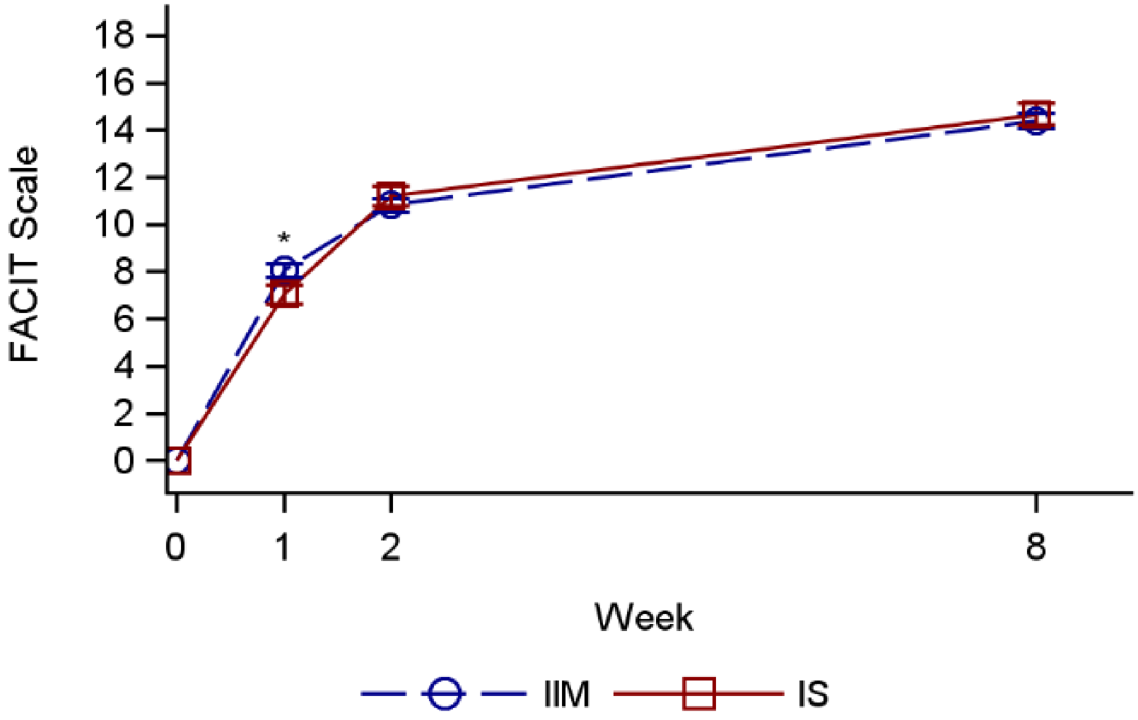

Estimates (mean and SE) from a mixed model with repeated measures with strata, treatment and time as factors, treatment*time and baseline value*time interactions and baseline value as covariate

IIM: Iron isomaltoside 1000/ferric derisomaltose, IS: Iron sucrose

*) p < 0.05, **) p 0.001-0.01, *** p < 0.001
